# Performance Degradation Mechanism of New Grouting Filling Material Under Goaf Erosion Environment

**DOI:** 10.3390/ma18225147

**Published:** 2025-11-12

**Authors:** Han Yang, Junwu Xia, Yujing Wang, Yu Zhou, Kangjia Song, Siyong Tan

**Affiliations:** State Key Laboratory of Intelligent Construction and Healthy Operation and Maintenance of Deep Underground Engineering, China University of Mining and Technology, Xuzhou 221116, China; yanghan2680@163.com (H.Y.); tb21030007b4@cumt.edu.cn (Y.W.); zh0uyu1006@163.com (Y.Z.); 18751281121@163.com (K.S.); tansy1999@126.com (S.T.)

**Keywords:** sulphate attack, chloride attack, grouting and filling material, erosion mechanism, microstructure characteristic, goaf management

## Abstract

This study aims to resolve the “secondary activation” challenge when erecting structures over goaf zones by employing a novel grouting and filling material. It delves into the performance degradation of the innovative ECS soil grouting filling material (ESGF material) within the goaf’s ionic erosion context. Erosion tests were performed on ESGF material specimens with varying mix designs to mimic the sulfate and chloride erosion scenarios commonly encountered in practical engineering. The macro-mechanical properties and microstructural changes of ESGF materials under ionic erosion environment were systematically investigated by various testing methods, such as unconfined compressive strength (UCS), SEM, XRD, TG, FTIR, and Raman. The findings indicate that both sulfate and chloride erosion lead to a reduction in the strength of the ESGF material. As erosion progresses, the specimens experience a mass increase followed by a decrease, with their strength exhibiting a consistent downward trend. In sulfate erosion conditions, the buildup of expansion product like ettringite (AFt) and thaumasite (TSA) inflicts substantial internal structural damage. Conversely, Friedel’s salt, the primary product of chloride erosion, exhibits relatively weaker expansiveness, and chloride concentration exerts a less pronounced effect on material degradation. Moreover, the cementitious material content and the proportion of quick-setting component play a significant role in determining the ESGF material’s resistance to erosion. By adjusting the quick-setting components ratio in response to changes in the water content of soft soil, the anti-ion erosion performance of solidified soil can be effectively enhanced. Notably, curing with a 5% sulfate maintenance could significantly improve the erosion resistance of ESGF material. This suggests that ESGF materials can be used without concern for curing issues in high-salinity environments during grouting. The research addresses the root cause of goaf subsidence while facilitating the recycling of solid waste, offering an environmentally friendly solution.

## 1. Introduction

**Figure 1:**
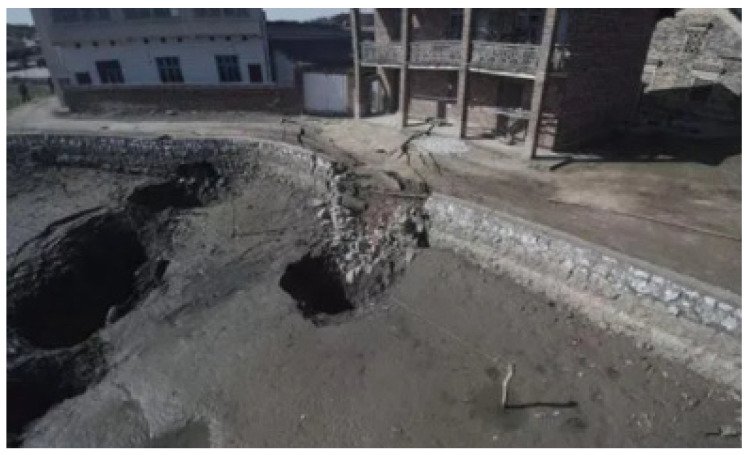
Surface subsidence in goaf areas.

In recent years, with the continuous development of the economy and modern society, energy has assumed a particularly strategic position. Coal resources remain the most reliable, stable, and important energy source in the short term [1]. However, large-scale mining of coal resources has led to frequent occurrences of surface subsidence in mining areas, as shown in Figure 1, a problem that has become increasingly severe in recent years [2]. According to incomplete statistics, the area affected by coal mining subsidence in China has already exceeded 2000 km^2^. This has seriously restricted the construction and planning of major infrastructure projects such as modern railways, airports, highways, water conservancy projects, and pipelines. It has also significantly reduced the ideal land area available for construction in mining regions and has had a major impact on the stability of mining areas and the well-being of the people [3,4,5]. Therefore, it is necessary to conduct an in-depth study on the issues of ecological environment and production safety impacts caused by mining subsidence, as well as the construction and management of goaf areas [6].

When constructing new buildings over goaf areas, factors like loads and external forces can jointly trigger the “secondary activation” of old goaf zones. To ensure the safety of constructions above goaf areas, it is essential to treat these zones [7,8,9]. Considering that the construction of the upper part of the goaf will produce a large amount of slag soil. Utilizing these construction wastes by solidifying them with a novel Expansion-Cementitious Compound Stabilizer (ECS) to produce new grouting filling materials for goaf treatment is a multi-benefit solution.

Once injected into goaf areas, the grouting filling material is susceptible to various factors like the high temperature and humidity, surrounding pressure, and mine water erosion within the goaf [10]. The chemical composition of mine water in some areas is shown in Table 1. Due to the high water content of the grouting material, mine water rich in sulfates can easily erode the internal structure of the filling body. This may eventually cause cracks inside the filling body [11], lead to the loss of supporting function, and render the filling ineffective and destabilize the goaf [10,12,13]. Therefore, the influence of ionic erosion should be considered during the filling process [14].

Since the 1990s, scholars have observed that sulfate erosion of cement and lime in the environment causes expansion, leading to swelling and damage in projects such as highways and railways [15,16,17,18]. The extant literature on the durability of cured soils is primarily focused on low moisture content soils used as roadbeds and other backgrounds [19,20]. However, there are few studies on the erosion performance of soft soils with high moisture content when used as grouting and filling materials. Cao et al. [21] used SP materials and cement to stabilize high-moisture dredged sludge (with water contents of 85%, 104%, and 124.5%). The study found that a higher dosage of binding materials (20% SP and 40% cement) was required to meet test conditions such as demolding. Feng et al. [22] research indicated that sulfoaluminate cement positively influenced the water stability of the sludge and the solidification of heavy metals. Additionally, oxychloride cement was also found to have a beneficial effect on the solidification process [23,24].

The resistance of materials to sulfate erosion is influenced by their mineral composition. Maria et al. [25] studied 20 soils with different mineral compositions after improvement and analyzed their sulfate erosion reactions and expansion under varying sulfate concentrations. They found that ettringite crystal formation is influenced by pore distribution, cement properties and dosage, mineral composition of the improved soil, and environmental factors. The pH value also affects the products of sulfate erosion of cement soil [26,27]. Studies found that ettringite and silicate gypsum crystals appear when the pH is above 11, and ettringite crystals gradually disappear to become amorphous when the pH is below 9.5. When the pH is between 12 and 13, hydrated calcium silicate reacts with sulfate ions to form thaumasite. Temperature also has a certain effect on sulfate erosion [28], Yang et al. [29] found that with the increase of temperature, the degree of deterioration of sulfate on cementitious materials decreases and then increases, and the degree of deterioration is lowest at about 10 °C. Additionally, some mineral admixtures like fly ash, slag, and silica fume can enhance the material’s resistance to ionic erosion. McCarthy et al. [30] studied the impact of fly ash on the sulfate erosion expansion performance of lime-stabilized soil, considering factors like quicklime dosage, fly ash dosage, and fly ash fineness. They found that sulfate erosion expansion increases with the total sulfate content in the lime-stabilized mixture but decreases with increasing fly ash dosage and fineness.

Overall, ionic erosion is mainly affected by the external environment (e.g., different erosion media, concentration, temperature, pH, etc.) and internal environment (e.g., pore space, sulfate and aluminate in raw materials, etc.). In addition, some mineral admixtures such as fly ash, slag powder, silica fume, etc., will also enhance the anti-ionic erosion performance of the materials to a certain extent [31,32,33,34,35]. The composition of the new ECS soil grouting filling material (ESGF material) includes clay, silicate cement, sulfoaluminate cement, lime, gypsum and additives, etc. Due to its complexity, the mechanical property degradation of ESGF material under sulfate and chloride erosion after being filled into goaf areas is unclear, making it necessary to systematically study its ionic erosion resistance.

## 2. Materials and Methods

### 2.1. Test Materials

The water used for the test was ordinary tap water from Xuzhou city, and the water temperature was 20–25 °C. The test soil was taken from the third clay layer of the pit at the site of the Student Innovation Training Center of China University of Mining and Technology, Nanhu Campus (No.1 Daxue Road, Tongshan District, Xuzhou City, Jiangsu Province, China), and the original clay was air dried, crushed, passed through a 2 mm sieve, dried, and then sealed and bagged as the test soil. The PH value, specific gravity, plastic limit, liquid limit, and particle size distribution of the test soil were measured, which are shown in Table 2.

### 2.2. Mineral Composition of Experimental Soil

**Figure 2:**
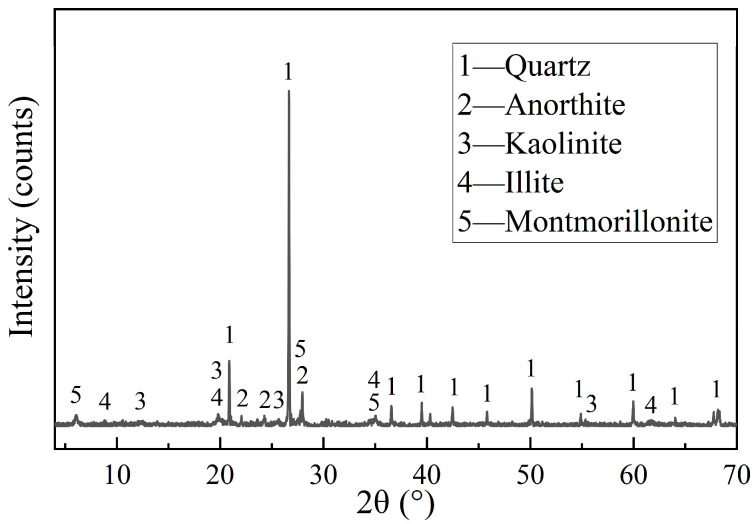
XRD graph of the test soil.

The mineral composition of the test soil was analyzed by XRD as shown in Figure 2, and the main mineral constituents were quartz, kaolinite, illite, and montmorillonite. The mass percentage of each oxide composition in the test soil was obtained by X-ray fluorescence (XRF) analysis and is shown in Table 3.

### 2.3. New ECS Soil Grouting Filling Material

The new ECS cementitious material has been authorized by the national patent, patent number CN201811390913.3 [36]. The new cementitious material is mainly composed of cementitious component and quick-setting component, in which the cementitious component is ordinary silicate P•O42.5 cement, and the quick-setting component consists of two parts, A material and B material, in which the specific composition is shown in Table 4.

### 2.4. Design of Test Mix Proportion

The soils used in the experiments were all prepared high-moisture soft soils with a water content of 100% to 150%, represented by the letter W. The letter E represents the dosage of the new ECS cementitious material, for example, E20 has a dosage of 20%, which is equivalent to a water cement ratio of 5:1. The letter P represents the proportion of quick-setting component in the binder composition, the swelling agent content in the binder composition was 0.3, 0.5, and 1.0. Due to the excessively high water–binder ratio (≈7:1), the OPC-only control exhibited severe bleeding and inadequate strength; therefore, erosion testing was not conducted. The curing method used was standard curing. In addition, considering the actual high salt-water environment in the goaf, YS is used to represent the 5% sulfate curing group. The specific mix ratios are shown in Table 5.

### 2.5. Erosion Schemes

The control group in the experiment was subjected to soaking in distilled water. The experimental groups were exposed to 5%Cl^−^, 10%Cl^−^, 5%SO_4_^2−^, 10%SO_4_^2−^, and the coupling of the two ions. The specific erosion schemes are presented in the Table 6.

### 2.6. Experimental Process

**Figure 3:**
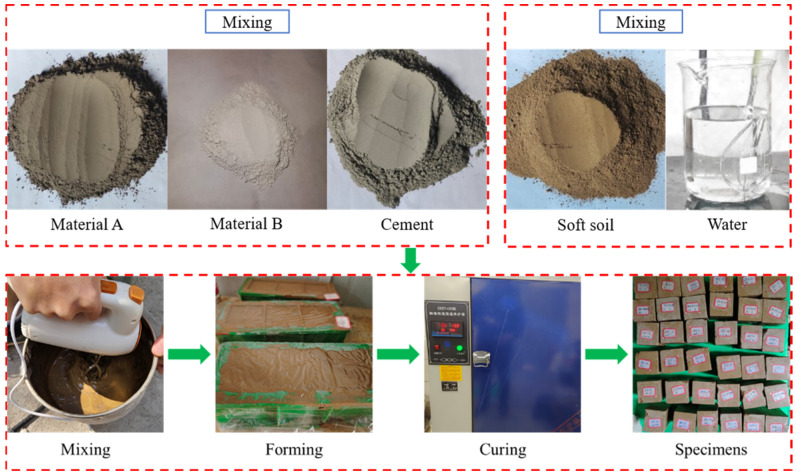
The pouring process of the specimens.

**Figure 4:**
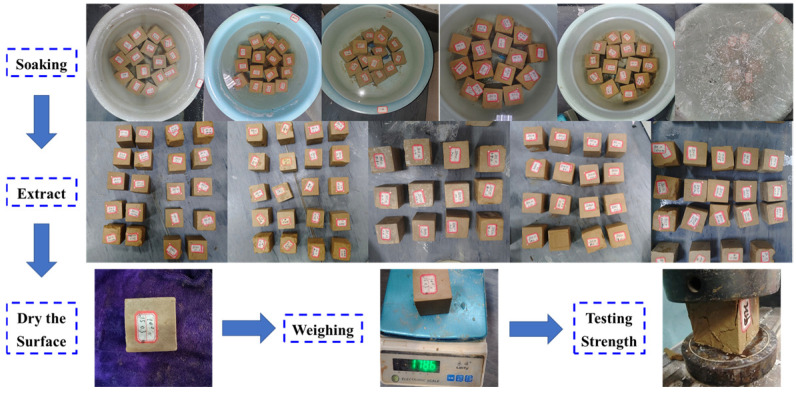
Ion erosion test process.

Weigh the A material, B material, cement, soil and water according to the proportion, add the weighed soil to the weighed water and mix it well; then, add the A and B material and cement, and mix it for 4 min by using a mixer. The mixed slurry was poured into 50 × 50 × 50 mm^3^ cubic specimen molds coated with Vaseline release agent, vibrated and shaped on a small vibrating table. Three specimens in each group were smoothed and covered with plastic film. After 1 day, all specimens were demolded, wrapped in plastic film and subjected to standard curing, as shown in Figure 3. In the specimen maintenance to 27 d when removed and soaked in water for 1 d, to reach 28 d maintenance age, dry the specimen surface and weighing; then, the specimen will be placed into the corresponding erosion environment for erosion. In order to prevent the effect of evaporation on the concentration of the erosion solution, the surface of the erosion container was coated with a film to prevent evaporation of the solution. At the age of 40, 80, and 120 days, the specimens were removed, dried, weighed, and subjected to a uniaxial compression test as shown in Figure 4.

## 3. Experimentation Results

### 3.1. Mass Variation

**Figure 5:**
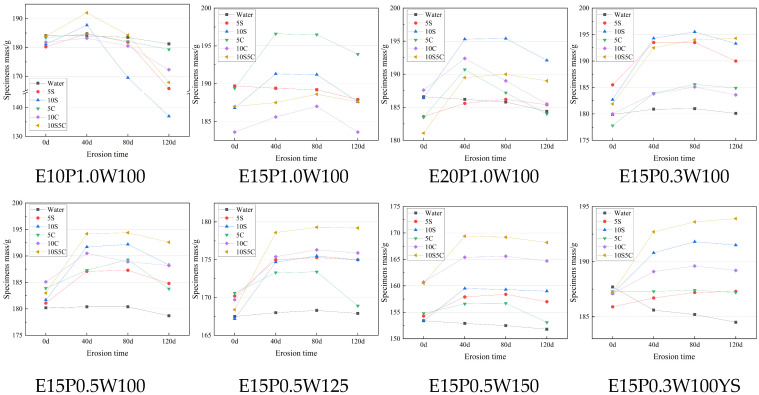
The variation of specimen mass with time.

The mass changes of specimens with different mix proportion after erosion are shown in Figure 5. It can be seen that in all groups of specimens in the control group of the clear water environment, the mass change is not obvious, while the other groups all appear in the mass of the first increase and then show a decreased trend. This also indicates that the hydration products in the specimens reacted with the salt solution at the beginning of the reaction, and that sulfate erosion produced products such as AFt, TSA, whereas salt erosion produced Friedel’s salt. Ionic erosion advances layer by layer, and as the erosion time increases, the amount of expansion product generation gradually increases and begins to fall off layer by layer. Specimen surface erosion is the most serious, and by the surface layer of erosion and expansion of the influence of the surface layer of erosion, in 120 d, the specimen surface is more obvious dissolution of the phenomenon of shedding, resulting in a degradation of the quality of the specimen.

With the increase in the dosage of cementitious materials, there is a significant increase in the quality of the specimen in each erosion environment erosion at 40 d and the overall trend of the first rise, and then there is a decline. At lower dosage (10%), the specimen structure was looser in texture and lower in strength, resulting in faster surface layer detachment at the later stage of each erosive environment (80–120 d), and the specimens had a substantial loss of quality. As the water content of the soft soil increased, the trend of the mass change of the specimens did not change significantly, and there was a small decreasing trend. This indicates that the curing effect of the new ESGF material is more satisfactory for soft soil with high water content, and it can still generate a dense AFt-CSH structure at higher water content.

The influence of the proportion of quick-setting component on the quality change of the material is relatively small, and with the increase of the proportion of quick-setting component, the quality change of the specimen in the sulfate erosion environment shows an overall decreasing trend. This is due to the fact that the incorporation of the quick-setting component introduces a large amount of Ca^2+^, which shares part of the Ca^2+^ consumption in sulfate erosion. The change of the maintenance condition from standard maintenance to 5% sulfate maintenance had considerable improvement on the mass loss of the material, and the ratio of the average mass change fraction of 5% sulfate maintenance to the standard maintenance specimen was 0.338:1. This was due to the fact that the solution provided enough SO_4_^2−^ to generate enough calcium alumina in the material to form a dense AFt-CSH structure, and therefore avoided the subsequent ionic in the large pores of the material. Concentrated erosion and mass loss was drastically reduced, which is also consistent with the strength change results below.

**Figure 6:**
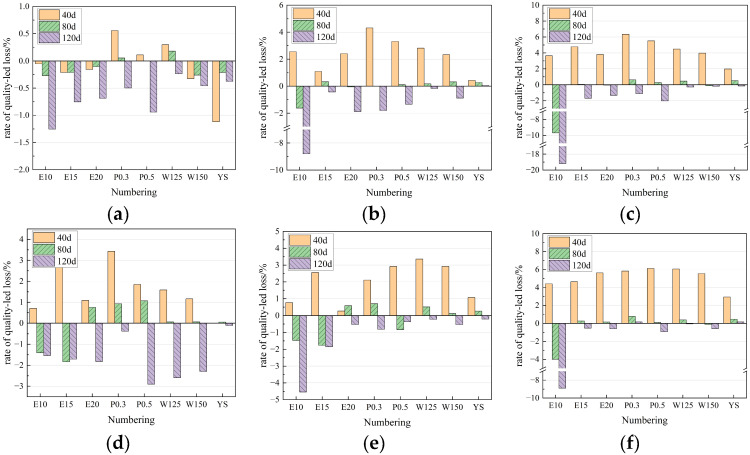
Change rate of specimen quality loss under different erosion environments. (**a**) Water; (**b**) 5S; (**c**) 10S; (**d**) 5C; (**e**) 10C; (**f**) 10S5C.

Figure 6 illustrates the mass loss rates of specimens across different erosion environments. In the environment of 5% and 10% chlorine salt erosion, the mass of the sample increased by 1.945% and 1.997% on average after 40 days of erosion. At 120 days of erosion, the average mass loss rate was 1.668% and 1.136%. It can be seen that the relationship between the sample and the concentration of erosion ions is not obvious in the environment of chlorine salt erosion. By 80 d, most specimens in chloride environments continue to gain mass, but groups (1) and (2), with lower pure quick-setting component content, exhibit substantial mass reduction. This may be due to the fact that the sulphoaluminate cement in the quick-setting component alone cannot form a dense structure without the incorporation of the cementitious component.

Under 5% and 10% sulfate erosion environment, the mass increase of erosion 40 d was 2.408% and 4.314%, which was basically consistent with the increase of sulfate ion concentration. The mass change was less than 1% after 80 days of erosion, and the mass decrease was 1.907 and 3.246% after 120 days of erosion, respectively. This explained that with the progressive layers of erosion, the specimen pore space gradually expanded and connected, until the surface gradually dissolved off, resulting in the generation rate of the erosion products in the specimen becoming gradually smaller than the dissolution of the specimen and the surface layer off, so that the test mass first increasing and then slightly decreasing.

In the coupled sulfate–chloride erosion environment, the mass increase at 40 d of erosion is higher than that of the same concentration of sulfate erosion group, while the mass changes at 80 d and 120 d are smaller. This may be due to the faster chloride ion transport rate at the beginning of the reaction, while the sulfate ions only reacted at a shallow level and were subject to less inhibitory effects on each other. With the increase of time, both sulfate and chloride ions reacted at all erosion depths of the specimens, and the two ionic reactions inhibited each other resulting in a lower rate of mass loss than single erosion.

### 3.2. Compressive Strength Change

**Figure 7:**
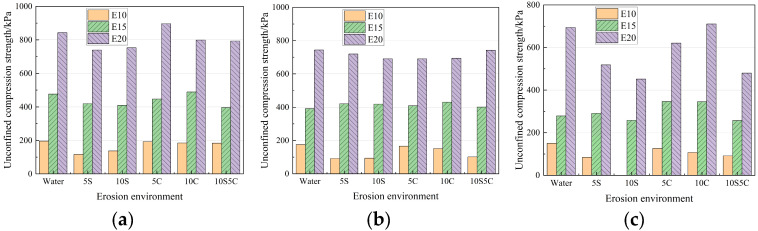
Strength of specimens with different dosage of cementitious materials after erosion. (**a**) 40 d; (**b**) 80 d; (**c**) 120 d.

The variation rule of strength after erosion of specimens with different dosage of cementitious materials is shown in Figure 7. Overall, the resistance of the specimens to chloride salt erosion is comparable to that of the water control group and is better than that of sulfate erosion. With the increase of the dosage, the average strength reduction rate in each erosion environment at 120 d was 48.13%, 32.73%, and 28.15%, respectively. It can be seen that with the increase of the dosage of the cementitious material, the material’s resistance to ionic erosion performance is gradually improved, and in the dosage of the dosage from 10% to 15% is more obvious. Considering cost-effectiveness, a 15% cementitious content is recommended for practical applications. The specimen in Figure 7c with a 10:1 water–cement ratio was not able to perform the compressive test after 120 d of erosion in a 10% sulfate–aggressive environment because the erosion was too severe.

**Figure 8:**
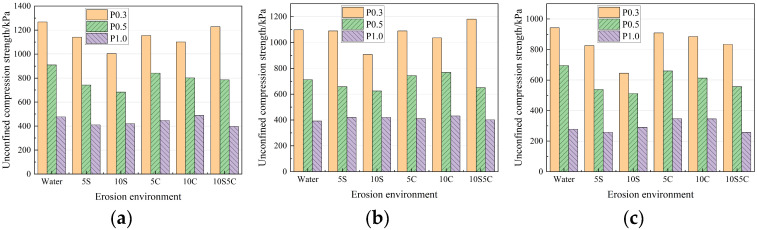
Strength of specimens with different quick-setting components after erosion. (**a**) 40 d; (**b**) 80 d; (**c**) 120 d.

The changed rule of the strength of specimens with different quick-setting components is shown in Figure 8. With the increase of the quick-setting component, the average strength discount rate in each erosion environment erosion 120 d was 26.97%, 25.08%, and 32.74%, respectively. It can be seen that the effect of ions on the strength of specimens with different proportions of quick-setting components showed a decreasing and then rising trend. The quick-setting components accounted for better (P0.5), and the specimen’s resistance to erosion was better. This is due to the fact that the hydration products in the quick-setting component have AFt, which has the effect of fixing water, and there is an optimum value provided that the water–cement ratio and water content remain unchanged.

**Figure 9:**
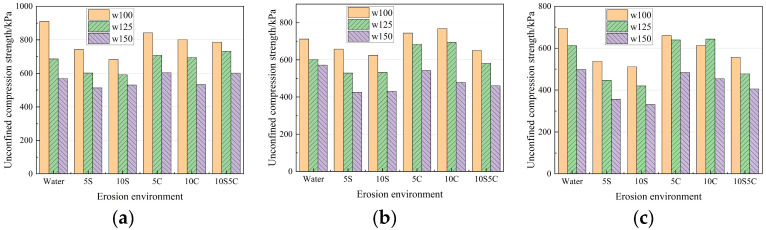
Strength of soft soil specimens with different moisture content after erosion. (**a**) 40 d; (**b**) 80 d; (**c**) 120 d.

The changed rule of strength after erosion of specimens with different soft soil water content is shown in Figure 9. With the increase of water content, the average strength discount rate at 120 d in each erosion environment is 25.08%, 19.58%, and 24.53%, respectively, which can be seen due to the new ESGF material in the higher water content, but still can generate a more dense AFt-CSH structure, so that the strength of each group of specimens is not much different. In terms of strength changes in different erosion environments, the order is as follows: chloride erosion ≈ clear water control group > coupled erosion > sulfate erosion.

**Figure 10:**
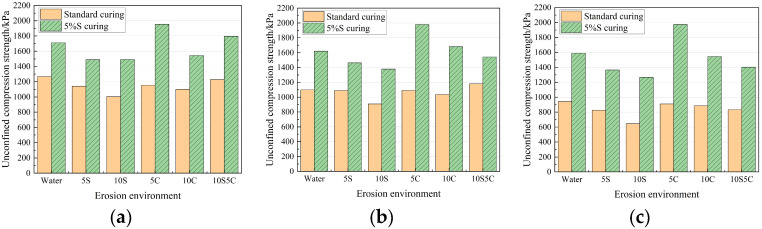
Strength of specimens under different curing conditions after erosion. (**a**) 40 d; (**b**) 80 d; (**c**) 120 d.

**Figure 11:**
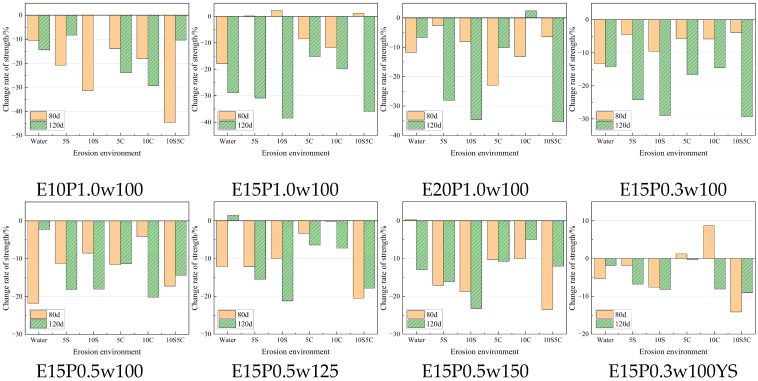
The variation law of specimen strength with erosion time.

As shown in Figure 10, the strength development of specimens cured under different conditions reveals notable enhancements for those subjected to 5% sulfate curing. When compared to specimens with identical mix proportions across various erosion scenarios, the average strength of the specimens after 40 d, 80 d, and 120 d erosion enhancement of 45.04%, 46.46% and 76.51%, respectively. It can be seen that the strength of the specimens after sulfate conditioning is not lower due to the longer relative immersion time, but rather the strength significantly increased, and the strength of the specimens in the later stages of erosion increased even more. This is due to the fact that the 5% sulfate solution provided enough SO_4_^2−^ to generate enough AFt with the lime gypsum in the quick-setting component and formed a dense calcium AFt-CSH structure with the cementitious component. As shown in Figure 11, the strength of the specimens generally decreases with longer erosion times. At 80 days, the strength loss is relatively low, and some specimens even exhibit slight strength gains due to pore-filling by expansion product. However, at 120 days, the 5S and 10S specimens (exposed to sulfate attack) show significant strength declines, while the 5C and 10C specimens (exposed to chloride attack) maintain a relatively higher strength. The strength loss under coupled erosion lies between that caused by sulfate and chloride ions alone.

The concentration of erosion ion also affects strength degradation. In sulfate environments, the strength loss increases with higher sulfate concentrations. For instance, the average strength loss at 5% and 10% sulfate concentrations is 27.53% and 36.39%, respectively. In contrast, chloride concentration has minimal impact on strength degradation, with only a 0.1% difference between 5C and 10C specimens. The degradation amplitude of the specimen in the coupled erosion environment is 34.14%, which is lower than the same concentration of sulfate erosion, in line with the mutual inhibition law of sulfate ion and chloride ion coupled erosion.

### 3.3. The Apparent Morphological Changes of the Specimen

**Figure 12:**
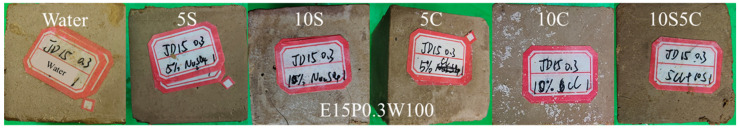
The apparent morphology of the specimen at the erosion time of 40 d.

As shown in Figure 12, after 40 days of erosion, specimens with the quick-setting component ratio of 0.3 exhibit surface cracking under 10% sulfate attack, with more cracks than under coupled 10% sulfate and 5% chloride attack. No significant cracks are seen in chloride or water environments. This indicates that the novel ESGF material is most vulnerable to sulfate erosion, followed by coupled erosion, and then chloride erosion.

**Figure 13:**
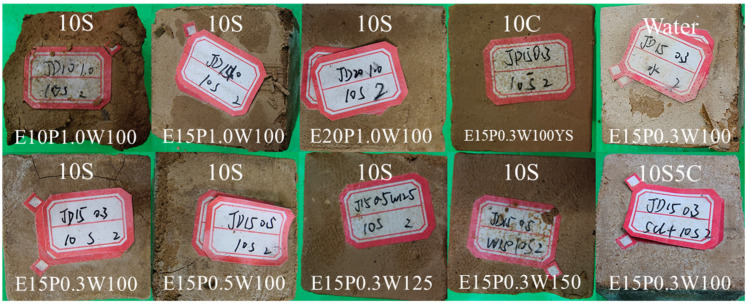
The apparent morphology of the specimen at the erosion time of 80 d.

As shown in Figure 13, after 80 days of erosion, specimens in the deionized water control group exhibit some surface scaling. In 10% sulfate environments, specimens with lower cementitious material content (10%) show pronounced surface and edge scaling. As cementitious material content increases, the damage pattern shifts to corner loss and surface layering. When the quick-setting component ratio is low, surface cracks appear; however, these can be prevented with proper quick-setting component content or sulfate curing. In coupled erosion scenarios, no surface cracks are observed, indicating a mutual inhibitory effect between chloride and sulfate ions. Higher water content has a minimal effect on surface erosion. Even at elevated water content, specimens show only surface erosion, not severe corner loss.

**Figure 14:**
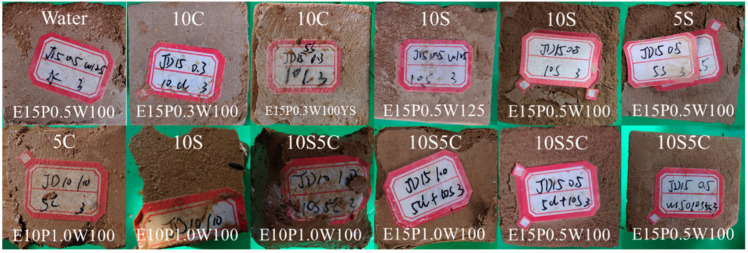
The apparent morphology of the specimen at the erosion time of 120 d.

As shown in Figure 14, after 120 days of erosion, specimens in chloride and water control groups exhibit distinct surface erosion marks. In sulfate environments, specimens show varying degrees of surface spalling and cracking, with higher sulfate concentrations exacerbating this damage. Notably, specimens in coupled erosion scenarios experience less surface degradation compared to those in sulfate-only environments of the same concentration. Specifically, the 10% dosage specimen, due to its low dosage and porous texture, suffers spalling in chloride environments, severe mudification in coupled chloride-sulfate environments, and undergoes such severe erosion in 10% sulfate environments that less than 50% of the specimen remains, rendering it unsuitable for compressive strength testing.

### 3.4. SEM Test Analysis

**Figure 15:**
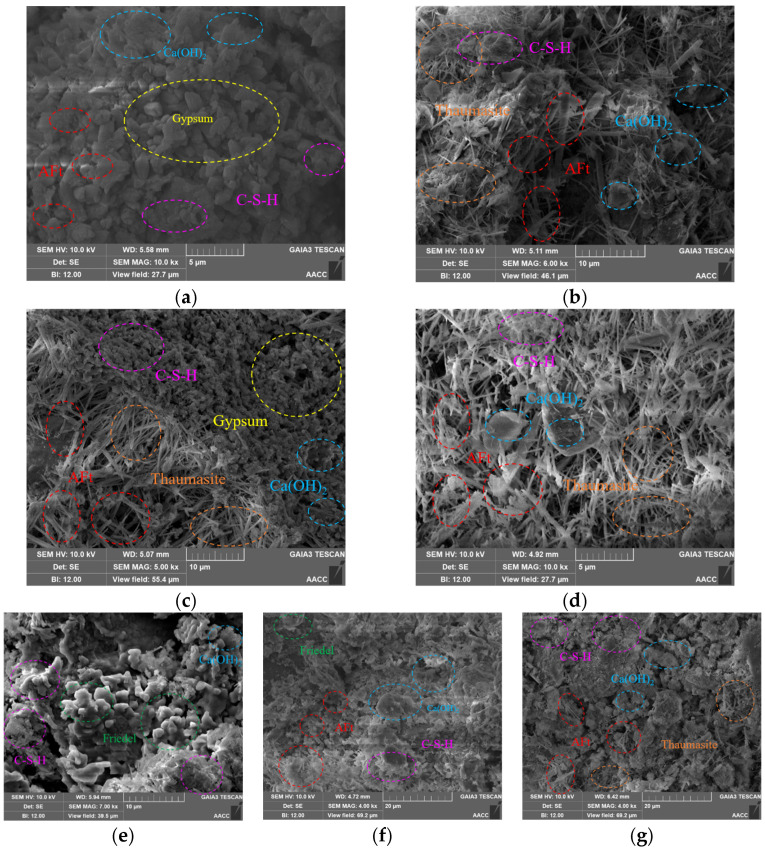
SEM diagram of the specimen after ion erosion. (**a**) 40d10s; (**b**) 120d10s; (**c**) W125120d10s; (**d**) E20120d10s; (**e**) 120d10c; (**f**) 120d10s5c; (**g**) P0.35syh10s.

As shown in Figure 15a,b, with the increase of erosion age, the amount of AFt in the specimen increases, along with the appearance of slender needle-like crystals, which are likely TSA, a product of sulfate erosion. Both AFt and TSA exhibit rod-like or columnar morphologies under SEM, but TSA has a more slender appearance with a higher aspect ratio [37].

As shown in Figure 15c,d, when the cementitious material content increases, the amount of AFt, CSH (CASH) and other hydration products increases. However, when the water content rises, the material’s porosity enlarges, leading to the formation of a large number of slender needle-like crystals within the pores.

From Figure 15e,f, it can be seen that under chloride ion erosion, the primary product is Friedel’s salt, while under coupled sulfate–chloride erosion, the formation of Friedel’s salt is significantly reduced. Additionally, the amount of AFt formed under coupled sulfate–chloride erosion is relatively less than that under pure sulfate erosion at the same sulfate ion concentration of 10%. This indicates a mutual inhibitory effect between chloride and sulfate ions during coupled erosion.

As shown in Figure 15g, after specimens are subjected to 5% sulfate curing and then exposed to sulfate erosion, the AFt and gypsum formed, along with other cement hydration products, create a denser network structure. This results in higher specimen density after curing and erosion, thus enhancing the material’s resistance to damage.

### 3.5. XRD Test Analysis

**Figure 16:**
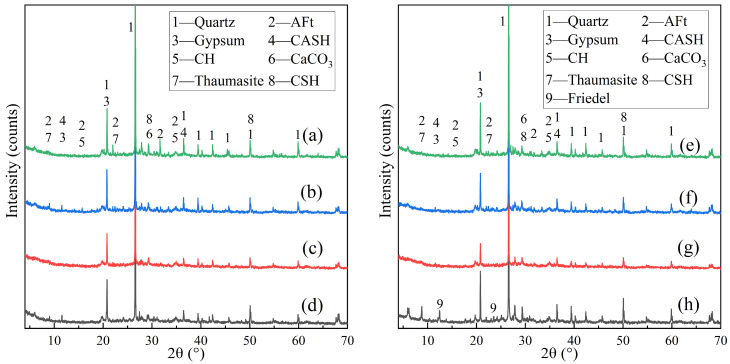
XRD spectrum of corroded specimen, where (**a**) E15P1.0W10040d10s; (**b**) E15P1.0W100120d10s; (**c**) E15P1.0W125120d10s; (**d**) E20P1.0W100120d10s; (**e**) E15P1.0w10010s5c; (**f**) E15P0.3w1005syh10s; (**g**) E15P0.3w10010s; and (**h**) E15P0.3w10010c.

As shown in Figure 16, the XRD patterns of specimens after erosion indicate that under a 10% sulfate attack, groups (a)–(d) exhibit weak CSH(CASH) gel, gypsum, and AFt peaks, along with faint Ca(OH)_2_ and CaCO_3_ peaks. However, due to the relatively low dosage of cementitious materials, these peaks are not prominent compared to the quartz peak. During sulfate erosion, TSA may form, but its peak overlaps significantly with that of AFt, necessitating Raman spectroscopy for TSA verification.

With the incorporation of cementitious components, a weak CSH gel peak appears. Yet, the poor crystallinity of CSH gel means that a weak peak does not imply low formation. In groups (e) and (h), which involve chloride ions, slight Friedel’s salt peaks are observed. In group (e), the AFt peak under coupled 10% sulfate and 5% chloride attack is weaker than in group (g) under sole 10% sulfate attack. This suggests that coupled erosion inhibits AFt formation, aligning with SEM findings.

### 3.6. TG-DTG Test Analysis

**Figure 17:**
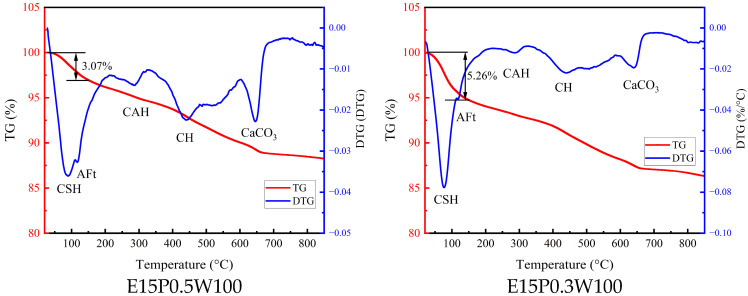
TG-DTG curve of the specimen corroded for 120 days in a 10% sulfate environment.

The TG-DTG curve of the specimens under 10% sulfate corrosion environment is shown in Figure 17, and the thermogravimetric results show that the mass-loss peak temperatures of different mixtures and curing ages occur at 86.5~140.0 °C, 228.5~237.7 °C, and 651.0~667.0 °C, corresponding to the dehydrations of amorphous CSH gels, AFt CAH, together with the decarbonation of CaCO_3_, respectively. Part of CaCO_3_ is generated by a carbonization reaction of Ca(OH)_2_, and the other part is a small amount of CaCO_3_ contained in the raw material B. In addition, it can be observed that as the proportion of quick-setting component decreases, the endothermic peaks of AFt and CAH decrease, while the amorphous CSH peak representing the hydration product of the cementitious component gradually increases.

### 3.7. FTIR and Raman Spectrum Analysis

**Figure 18:**
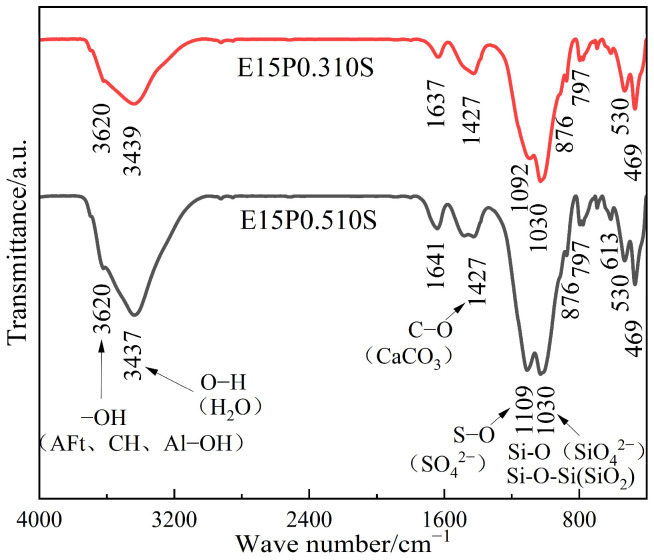
FTIR spectra of ESGF material after 120 days of erosion.

As shown in Figure 18, in the FTIR spectrum of the eroded specimens are as follows: the absorption bands at 3620 cm^−1^ are attributed to the stretching vibrations of Al-OH in kaolinite and the -OH in AFt and CH crystals. The bands near 3437 cm^−1^ and 1641 cm^−1^ correspond to the stretching and bending vibrations of O-H in the crystallization water and pore adsorbed water of CSH gel, AFt, and AFm crystals. The absorption bands near 1109 cm^−1^ and 613 cm^−1^ are assigned to the antisymmetric stretching and asymmetric bending vibrations of S-O in SO_4_^2−^ ions. The bands near 1030 cm^−1^ and 530 cm^−1^ are mainly caused by the overlapping antisymmetric stretching vibrations of Si-O-Si in quartz, and Si-O in kaolinite and CSH gel.

Since the specimens are predominantly soil, the weak absorption bands of Si-O in kaolinite and Si-O-Si in quartz make it difficult to observe the six-coordinated octahedral structure [Si(OH)_6_]^2–^ of TSA in the spectrum at 500 cm^−1^, 670 cm^−1^, and 750 cm^−1^, necessitating Raman spectroscopy for further analysis of TSA formation.

With the increase of the substitution rate of quick-setting component in cementitious material, the absorption bands of S-O in SO_4_^2−^ ions and O-H in crystallization water and pore adsorbed water both intensify, indicating an increase in AFt crystal generation. AFt crystals, known for their strong water absorption due to containing abundant crystallization water and adsorbing large amounts of free water in their structural gaps, show an increasing trend in quantity as the substitution rate of quick-setting component in cementitious material rises, as evidenced by the enhanced relevant absorption bands.

**Figure 19:**
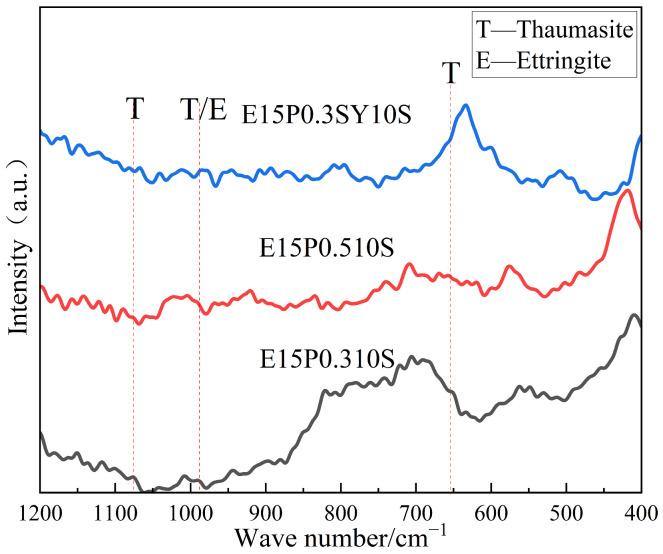
Raman spectra of a new ESGF material after 120 days of erosion.

For TSA, accurate quantification is notably challenging; XRD signals are weak and often overlap with AFt; SEM micrographs of TSA resemble those of AFt, with both exhibiting well-defined rod-like (acicular) crystals; FTIR absorption bands of TSA are of low intensity and cannot be distinctly identified in the spectra. In order to verify the generation of TSA, an erosion product during the erosion process, Raman spectral analysis was carried out as shown in Figure 19. It can be seen that the six-coordinated Si(OH)_6_^2−^ characteristic peak in TSA is at around 658 cm^−1^, the carbonate characteristic peak is at 1076 cm^−1^, and the sulfate characteristic peaks in TSA and calcite are present at 990 cm^−1^, respectively. The peak intensity of each erosion product is low because clay is still dominant in the new ESGF material. However, it can still be seen that after 120 d of sulfate erosion, there is some TSA generation in each group of specimens.

## 4. Discussion

Under goaf conditions, confining pressure, temperature, and fluctuations in high-salinity groundwater level may affect the durability of grouting backfill materials. Since confining pressure generally has a beneficial effect on durability, it was excluded. Although elevated temperature can accelerate ion transport and reactions, the subsurface temperature field is relatively stable and its net impact is limited. Given the persistently high underground humidity, the influence of saline water-level fluctuations is weaker than surface wet–dry cycling. Accordingly, these factors were not treated as experimental variables.

Macro-testing methods like mass loss and UCS, along with micro-testing methods such as SEM, XRD, TG-DTG, FTIR, and Raman, have varying sensitivity to novel ESGF materials with different mix proportions in diverse erosion environments. No single testing method is sufficient for effective evaluation. The results of various test methods complement and verify each other, the corrosion products and microscopic morphology of ESGF materials in sulfate and chloride environments were further determined, and the mechanism of the influence of ions on the strength of ESGF materials was further explored.

**Figure 20:**
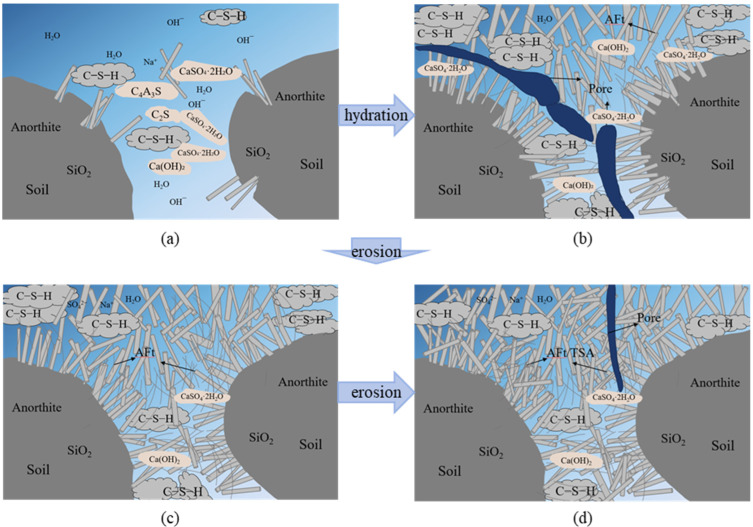
The schematic diagram of ESGF materials subjected to sulfate attack. where (**a**) Newly poured specimens; (**b**) Test specimens during maintenance; (**c**) Erosion of early stage specimens; (**d**) Erosion of late stage specimens.

The hydration products of novel ESGF materials are mainly CSH gel and AFt crystals, along with CH crystals, CAH crystals, and CaCO_3_. Their strength primarily depends on CSH gel, AFt crystals, and CaCO_3_. Needle like AFt crystals form via the reaction of gypsum with tricalcium aluminate (C_3_A) and tetracalcium aluminoferrite (C_4_AF) in cement [38,39]. Ultimately, CSH gel cements soil particles and the AFt crystal network into a relatively dense structure, as shown in Figure 20b.

In the early stages of sulfate erosion, the sulfate concentration is relatively high due to accelerated testing considerations. In novel ESGF materials, gypsum crystals are formed in addition to AFt [Figure 15a]. This is why the specimen mass increases early in sulfate erosion. The reaction equation is as follows:

(1) $${\mathrm{Ca}}^{2+}+{\mathrm{SO}}_{4}^{2-}+2H_{2}O\to {\mathrm{CaSO}}_{4}\cdot 2H_{2}O$$

However, as the rate of sulfate erosion increases, the erosion process will not only yield AFt crystals directly, but gypsum will also undergo further reaction to produce the erosion product AFt crystals [Figure 15b]. At this time, accompanied by the phenomenon of peeling off the surface of the specimen after erosion, the mass of the specimen will appear to remain unchanged, as shown in Figure 20c. The reaction equation is presented below:

(2) $$\begin{array}{c} 3({\mathrm{CaSO}}_{4}\cdot 2H_{2}O)+4\mathrm{CaO}\cdot {\mathrm{Al}}_{2}O_{3}\cdot 12H_{2}O+14H_{2}O\to \\ 3\mathrm{CaO}\cdot {\mathrm{Al}}_{2}O_{3}\cdot 3{\mathrm{CaSO}}_{4}\cdot 32H_{2}O+\mathrm{Ca}{\left(\mathrm{OH}\right)}_{2} \end{array}$$

As sulfate erosion time increases, Raman spectroscopy detects TSA formation. The formation mechanism is outlined as follows: if Si^4+^ in the CSH gel replaces A1^3+^ in the caliche, and [SO_4_^2−^ + CO_3_^2−^] replaces [SO_4_^2−^ + H_2_O] in the AFt, carbon, sulfur, calcium, TSA will be generated. The reaction equation is shown below:


(3) $$\begin{array}{c} {\mathrm{Ca}}_{6}{\left[{\mathrm{Al}(\mathrm{OH})}_{6}\right]}_{2}{\left({\mathrm{SO}}_{4}\right)}_{3}\cdot 26H_{2}O+{\mathrm{Ca}}_{3}{\mathrm{Si}}_{2}O_{7}\cdot 3H_{2}O+{\mathrm{CaCO}}_{3}\\ +{\mathrm{CO}}_{2}+xH_{2}O\to {\mathrm{Ca}}_{3}{\left[{\mathrm{Si}(\mathrm{OH})}_{6}\right]}_{2}{\left({\mathrm{CO}}_{3}\right)}_{2}{\left({\mathrm{SO}}_{4}\right)}_{2}\cdot 24H_{2}O\\ +{\mathrm{CaSO}}_{4}\cdot 2H_{2}O+{\mathrm{Al}}_{2}O_{3}\cdot xH_{2}O+3{\mathrm{Ca}(\mathrm{OH})}_{2} \end{array}$$

When A13+ in AFt is replaced, it is released back into the ESGF material’s pore solution, forming new AFt. This new AFt then transforms into TSA. In consideration of the presence of Si4+ and abundant AFt in the novel ESGF material, it is postulated that AFt will undergo further transformation to yield calcium TSA. Yet, as erosion deepens, CSH gel in the specimen is progressively consumed from the surface inward. As a result, the specimen experiences considerable surface spalling and mudification, leading to a reduction in its mass. With the deepening of erosion, the formation of expansion products also increases, and finally expansion cracks will occur in the shallow layer of the sample, resulting in a gradual decrease in the strength of the sample, as shown in Figure 20c.

The chloride ion concentration exerts a lesser effect on the strength degradation of the specimen. This is attributed to the low expansiveness of Friedel’s salt, a product of chloride erosion, and the pore-blocking effect of the generated Friedel’s salt, which collectively slow down the chloride ion erosion rate and reduce the depth of free chloride ion penetration. The reaction equation is shown below:

(4) $$\begin{array}{c} 3\mathrm{CaO}\cdot {\mathrm{Al}}_{2}O_{3}\cdot {\mathrm{CaSO}}_{4}\cdot {12H}_{2}O\;(\mathrm{AFm}){+2\mathrm{Cl}}^{-}\to \\ 3\mathrm{CaO}\cdot {\mathrm{Al}}_{2}O_{3}\cdot {\mathrm{CaCl}}_{2}\cdot {10H}_{2}O\;(\mathrm{Fs}){+\mathrm{SO}}_{4}^{2-}{+2H}_{2}O \end{array}$$

In the complex ionic environment where sulfate ions and chloride ions co-exist, the two ions exist in a competitive relationship, and there is a mutual inhibition relationship on the erosion of the new ESGF materials. This conclusion can also be drawn based on the magnitude of the decrease in strength. The reasons are as follows: (1) Chloride ions migrate faster than sulfate ions in cementitious materials, enabling them to penetrate deeper into the material. Consequently, coupled erosion from both ions is primarily restricted to the surface and shallow regions. Moreover, the pore-blocking effect of Friedel’s salt partially mitigates the erosion rate [40]. (2) In the presence of two types of ions within an environment, the sulfate ion assumes a predominant role. In the presence of both ions within the environment concurrently, a reciprocal inhibition phenomenon occurs between sulfate and chloride erosion. This inhibition is predominantly associated with the presence of sulfate erosion product AFt and chloride erosion product Friedel’s salt within the erosion reaction process. These elements are required to participate in the process as Ca2+.

While this study has explored the ionic erosion resistance of ESGF materials by mimicking the high-salt water conditions in goaf areas and discovered that sulfate curing can substantially boost their resistance to ionic erosion. However, the surrounding pressure, high temperature and high humidity of grouting and filling in the actual project were not taken into account, and the effects of these factors coupled with ionic erosion on ESGF materials are not clear. Future research should focus on these aspects to achieve a more thorough understanding of how goaf environments affect ESGF materials.

## 5. Conclusions

This study investigates the ionic erosion resistance of novel ECS soil grouting filling materials (ESGF materials) and filling material tailored for goaf conditions with high salinity. Through UCS, SEM, XRD, TG, FTIR and Raman tests, the anti-ionic erosion performance of eight ESGF materials was systematically investigated under six sulfate and chloride erosion environments, as well as the changing rules of erosion products and microscopic morphology after erosion. Reaching the following conclusions:

(1) ESGF materials experience performance degradation under ionic erosion with a clear severity order of aggressiveness: SO_4_^2−^ > SO_4_^2−^ + Cl^−^ coupling erosion > Cl^−^. In the early stages, the reaction between hydration products and salt solutions generates expansive erosion products that fill pores. However, as erosion intensifies, the specimen structure degrades and falls off, leading to mass loss and strength reduction.

(2) The erosion resistance of ESGF materials is governed by mix design. Increasing the cementitious binder content enhances durability, while an intermediate proportion of the quick-setting component is most effective. The optimal proportion depends on the water content of the soft soil, providing practical guidance for performance-based mix design in the field.

(3) A total of 5% sulfate curing has a significant improvement effect on the anti-ionic erosion performance of ESGF materials. Compared to standard curing, specimens cured with 5% sulfate show a 76.51% increase in average strength after 120 days of erosion. This is attributed to the promotion of AFt formation during sulfate curing, creating a more compact AFt-CSH structure and thereby effectively improving the material’s erosion resistance. This finding provides an important theoretical basis for the use of ESGF materials in practical engineering to directly fill the working conditions of the mining zone containing high salt mine water.

(4) Sulfate erosion primarily generates AFt and TSA, whose formation and accumulation within the specimens lead to pore enlargement and structural loosening. In contrast, chloride erosion mainly produces Friedel’s salt, which causes relatively less damage to the specimen’s microstructure.

## Figures and Tables

**Table 1:** Hydrochemical composition of mine water in some mining areas.

Parameters	Huaiyuan Hengyuan Coal Mine	Yulin Coal Mine, Shaanxi	Hami Mining Area, Xinjiang	Guizhou Coal Mine
pH	7.86	7.91	7.73	4.41
SO_4_^2−^	1529	901.61	705.07	2008
Cl^−^	134.5	337.22	480.85	14.85
HCO_3_^−^	328.9	-	212.60	91.32
NO_3_^−^	-	4.58	-	-
Na^+^K^+^	355.6	534.73/7.51	553.11	66.21/5.67
Ca^2+^	173.5	157.24	104.80	232.4
Mg^2+^	205.5	26.95	31.38	78.44
Fe	-	0.2	-	241.9
Mn	-	-	-	4.27
Al	-	-	-	34.88

**Table 2:** Indicators of the characteristics of the soil used for the test.

Indicator	pH	Specific Gravity (GS)	Plastic Limit (WP)	Liquid Limit (WL)	Clay (<0.002 mm)	Silt (0.002–0.075 mm)	Sand (0.075–2 mm)
value	7.92	2.75	24.10%	44.20%	9.15%	80.28%	10.57%

**Table 3:** Composition of oxides in experimental soil.

Chemical Composition	SiO_2_	Al_2_O_3_	CaO	SO_3_	Fe_2_O_3_	MgO	K_2_O	TiO_2_	Na_2_O	Others
(%)	64.47	15.86	1.22	0.02	5.72	1.32	2.14	0.8	0.55	0.03

**Table 4:** Quick-setting components of new high water material.

	Material A		Material B	
Main Composition	3CaO·3Al_2_O_3_·CaSO_4_ 2CaO·SiO_2_ 4CaO·Al_2_O_3_·Fe_2_O_3_	Suspension agent Retarder Dispersant	CaSO_4_·2H_2_O CaO	Suspension agent Early strength agent Dispersant

**Table 5:** Mix ratio and slump of shield residues concrete.

Numbering	Short Name	Soil Moisture %	w/b	Expansion Component Content/%	Maintenance Conditions
(1)	E10P1.0w100	100	10:1	1.0	Standard Curing
(2)	E15P1.0w100	100	7:1	1.0	
(3)	E20P1.0w100	100	5:1	1.0	
(4)	E150P0.3w100	100	7:1	0.3	
(5)	E150P0.5w100	100	7:1	0.5	
(6)	E150P0.5w125	125	7:1	0.5	
(7)	E150P0.5w150	150	7:1	0.5	
(8)	E20P0.3w100YS	100	7:1	0.3	5% sulfate curing

**Table 6:** Erosion schemes.

Influencing Factors	Factor Level Value
SO_4_^2−^ Content	0%, 5%, 10%
Cl^−^ Content	0%, 5%, 10%
Coupled Erosion	10%SO_4_^2−^ + 5%Cl^−^
Corroded age	40 d, 80 d, 120 d

## Data Availability

The original contributions presented in this study are included in the article. Further inquiries can be directed to the corresponding author.
